# How accurate and statistically robust are catalytic site predictions based on closeness centrality?

**DOI:** 10.1186/1471-2105-8-153

**Published:** 2007-05-11

**Authors:** Eric Chea, Dennis R Livesay

**Affiliations:** 1 Department of Biological Sciences, California State Polytechnic University, Pomona, CA 91768, USA; 2 Department of Computer Science and Bioinformatics Research Center, University of North Carolina at Charlotte, Charlotte, NC 28223, USA

## Abstract

**Background:**

We examine the accuracy of enzyme catalytic residue predictions from a network representation of protein structure. In this model, amino acid α-carbons specify vertices within a graph and edges connect vertices that are proximal in structure. Closeness centrality, which has shown promise in previous investigations, is used to identify important positions within the network. Closeness centrality, a global measure of network centrality, is calculated as the reciprocal of the average distance between vertex *i *and all other vertices.

**Results:**

We benchmark the approach against 283 structurally unique proteins within the Catalytic Site Atlas. Our results, which are inline with previous investigations of smaller datasets, indicate closeness centrality predictions are statistically significant. However, unlike previous approaches, we specifically focus on residues with the very best scores. Over the top five closeness centrality scores, we observe an average true to false positive rate ratio of 6.8 to 1. As demonstrated previously, adding a solvent accessibility filter significantly improves predictive power; the average ratio is increased to 15.3 to 1. We also demonstrate (for the first time) that filtering the predictions by residue identity improves the results even more than accessibility filtering. Here, we simply eliminate residues with physiochemical properties unlikely to be compatible with catalytic requirements from consideration. Residue identity filtering improves the average true to false positive rate ratio to 26.3 to 1. Combining the two filters together has little affect on the results. Calculated p-values for the three prediction schemes range from 2.7E-9 to less than 8.8E-134. Finally, the sensitivity of the predictions to structure choice and slight perturbations is examined.

**Conclusion:**

Our results resolutely confirm that closeness centrality is a viable prediction scheme whose predictions are statistically significant. Simple filtering schemes substantially improve the method's predicted power. Moreover, no clear effect on performance is observed when comparing ligated and unligated structures. Similarly, the CC prediction results are robust to slight structural perturbations from molecular dynamics simulation.

## Background

The accurate and robust prediction of protein functional sites from sequence and/or structure remains an open problem in bioinformatics [1]. Despite the limitations of current methodologies, several sequence and structure-based approaches have recently become popular [2]. Most of these approaches rely on an underlying multiple sequence alignment and attempt to uncover some type of feature conservation therein [3] (i.e. residues that are conserved across the alignment [4-6]). Arguably, evolutionary tracing has become the most widely used method for computational prediction of protein functional sites [7]. The Evolutionary trace (ET) approach begins with an alignment and corresponding phylogeny. The method searches for all alignment positions that recapitulate the overall phylogeny. While ET is fundamentally a sequence-based scheme, the standard application of the approach uses structural clusters of trace residues to identify functional regions [8-10]. Several other related methods that rely on an underlying alignment plus representative structure have proven useful as well [11-14]. Conversely, we have introduced a phylogenetic motif-based method that is similar *in spirit *to ET, although it is specifically designed to rely solely on sequence information [15-17].

The literature also contains a host of functional site prediction strategies that are explicitly designed to not rely on a phylogeny [18]. These approaches are useful when too few sequences are available to generate a representative description of familial diversity. While their theoretical foundations vary considerably, most rely solely on structure or a structure + alignment combination. For example, Gutteridge et al. recently developed a neural network approach to predict catalytic sites [19]. *Catalytic *sites are defined by residues directly involved in the enzyme-mediated reaction mechanism, which generally constitute a subset of all *functional *residues. The neural network input of Gutteridge et al. includes both structural and alignment descriptors, and is able to correctly predict the active site in >69% of the cases examined. The ability to rigorously benchmark the approach is based on comprehensive databasing and exhaustive manual curation of catalytic residues from the literature [20] by the same group. This tour de force has led to the Catalytic Site Atlas (CSA) [21], which contains approximately 600 different proteins with experimentally validated catalytic residues.

Other common catalytic site prediction methods are based on Poisson-Boltzmann continuum electrostatic theory [22]. Elcock has observed that functional residues tend to have increased electrostatic strain energy [23], meaning that stabilization occurs on mutation. While the approach utilizes sophisticated Poisson-Boltzmann continuum theory, the underlying rationale is based on straightforward evolutionary arguments. The naïve description of protein evolution is that nature solely optimizes structural stability at each residue. However, catalytic and other important residues have functional constraints imposed upon them, meaning that while mutation might be stabilizing, it can occur at the expense of functional proficiency. The detangling of stability and functional evolutionary pressures is examined more thoroughly by Cheng et al. using all-atom protein design [24]. Analogous to the electrostatic strain energy approach, the THEMATICS approach uses Poisson-Boltzmann-based pKa calculations to look for residue titration curves that do not follow Henderson-Hasselbalch [25]. The method looks for titration curves of partially charged residues that are flat over a wide pH range. Similarly, we have demonstrated that a large pKa shift from the null model (aqueous) value can be indicative of catalytic residues [26,27]. However, the prediction accuracy of this approach is lessened because many structurally important residues (i.e. residues involved in a salt bridge) also have significant pKa shifts.

Network models have also been used with success in predicting protein functional and/or catalytic residues. Instead of representing protein structures as a Cartesian collection of atoms, network models recast protein structures as topological graphs [28-31]. The most common of these methods are based on protein structure contact maps, where each vertex of the graph represents an α-carbon and edges connect vertices within some distance cutoff (generally 6–9 Å). Once the graph is complete, a variety of topological metrics can be used to predict functional residues from it, including: centrality [32,33], valency [32] and sub-graph conservation [34]. Despite growing consensus concerning the utility of these methods, a robust assessment of their prediction accuracy remains to be completed. Amitai et al. [32], Thibert et al. [33] and del Sol et al. [35] examine the ability of residue centrality to predict catalytic and/or functional sites within datasets of 178, 128 and 46 proteins, respectively. The results from these studies are encouraging. Moreover, they show that combining centrality within other metrics improves predictive power. For example, Amitai et al. demonstrates that combining centrality with solvent accessibility substantially improves accuracy, whereas both Amitai et al. and Thibert et al. demonstrate that including residue conservation improves results.

In this report, we investigate the accuracy and statistical significance of closeness centrality (CC) functional residue predictions, which has previously been shown to be the best of several different network centrality scores (i.e. valency, betweenness, etc.) [32,33]. Primarily, our investigation is based on SCOP [36] superfamily-filtered protein chains (which represents 283 unique SCOP superfamilies) from the CSA. Based on observed accuracies, CC is demonstrated to be a viable prediction scheme. Our results are inline with previous investigations, but are more significant due to dataset size and composition since we control for structural redundancy. A second distinction of this work is that instead of focusing on the entire range of true to false positive rates, as done by previous investigations, we concentrate on the very best CC scores. By focusing only on the top five scoring residues, we are able to evaluate the ability of the model to provide insight that provides a reasonable number of experimentally testable predictions. In all cases, our predictions correspond to false positive rates below 1.6%. The performance of the method is improved substantially by considering only residues that are not completely inaccessible to solvent. We further demonstrate that filtering the predictions based solely on amino acid identity substantially improves predictive power even more than filtering by solvent accessibility.

## Theoretical background

Throughout this report, the vertices within each graph correspond to α-carbons. Edges connect two α-carbons within 8.5 Å of each other. While slightly less complicated than methods based on all-atom pair distances, the simpler model results in a noticeable computational speedup that significant when analyzing a dataset the size of ours. A cursory comparison of the two networks indicates that the resultant predictions are qualitatively similar (results not shown). The common threshold of 8.5 Å is used because it best approximates the average sidechain size. Closeness centrality (CC), a global centrality metric, is used to determine how critical each vertex (residue) is in maintaining the small-world behavior of the graph. CC is calculated by:

CCi=Np∑jLij
 MathType@MTEF@5@5@+=feaafiart1ev1aaatCvAUfKttLearuWrP9MDH5MBPbIqV92AaeXatLxBI9gBaebbnrfifHhDYfgasaacH8akY=wiFfYdH8Gipec8Eeeu0xXdbba9frFj0=OqFfea0dXdd9vqai=hGuQ8kuc9pgc9s8qqaq=dirpe0xb9q8qiLsFr0=vr0=vr0dc8meaabaqaciaacaGaaeqabaqabeGadaaakeaacqqGdbWqcqqGdbWqdaWgaaWcbaGaeeyAaKgabeaakiabg2da9maalaaabaGaemOta40aaSbaaSqaaiabdchaWbqabaaakeaadaaeqbqaaiabdYeamnaaBaaaleaacqWGPbqAcqWGQbGAaeqaaaqaaiabdQgaQbqab0GaeyyeIuoaaaaaaa@3BA9@

where N_p _is the total number of vertices in the graph and L_ij _is the shortest path (geodesic distance) between vertices *i *and *j*. The shortest path is simply the minimum of all possible paths between residues *i *and *j*. As normally done in protein structure networks, edges are not weighted, making the shortest path simply an integer count of the number of edges separating *i *and *j*. It should be noted that N_p _(a constant within each protein) has no effect on our observed results since we are only using CC to rank the residues, meaning the inverse of shortest path sum solely establishes which residues are ultimately predicted. Nevertheless, we employ CC here to be consistent with previous investigations.

## Results and discussion

### Probability density functions

**Figure 1:**
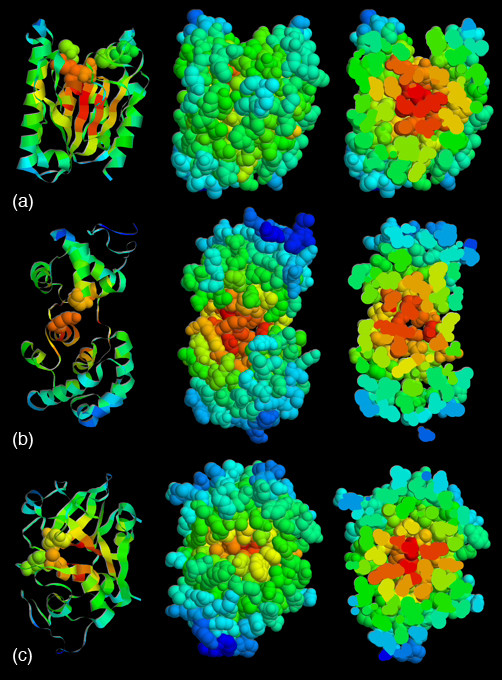
Closeness centrality is mapped to structure for: (a) *P. purpurogenum *Acetylxylan esterase (1BS9), (b) *e. coli *Endonuclease III (2ABK) and (c) *C. papaya *papain (9PAP). In all three cases, the most central residues occur near the center of the structure, which do not correspond to the catalytic residues. In each column, red indicates the most central residues; whereas blue indicates the least. In the column on the left, catalytic residues are shown in spacefill. In the center column, all atoms are displayed; in the column on the right, the structure has been sliced in half to highlight the most central residues within the hydrophobic core.

**Figure 2:**
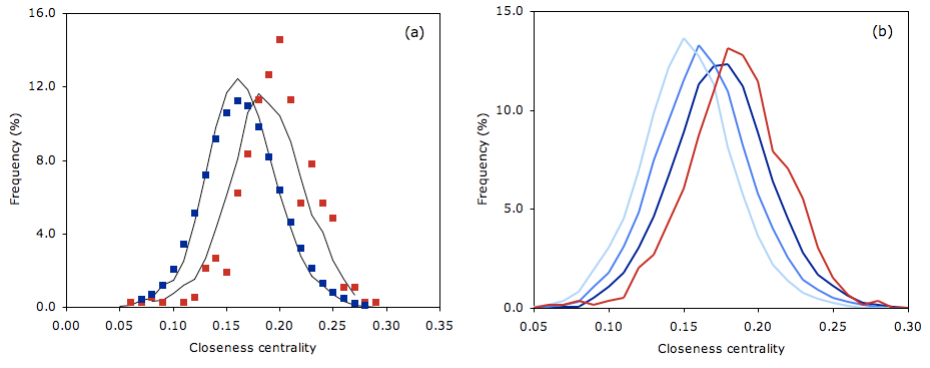
(a) Probability density function of the closeness centrality scores for all catalytic (red) and all noncatalytic (blue) residues. The distributions are scaled such that the integral of each is equal to one. The solid line trend line is simply a running average meant to help guide the eye. The average values for the two distributions are 0.16 and 0.19, which is a statistically significant distinction (for a two-sample t-test: t = 2.0; p = 1.6E-73; sample size = 7,373). The overlap of the two distributions across the histogram of sampled data is 59.5%. (b) Probability density functions for all catalytic sites (red line) compared to the distributions of all at three different solvent accessibility levels. Each distribution is scaled such that its integral is one. Statistics describing the distribution comparisons are provided in Table 1.

Mapping CC to structure clearly indicates that residues with high centralities are most likely to occur within the protein core. As is the case in the three examples shown in Fig. 1, catalytic residues frequently do not correspond to the most central residues. Nevertheless, Fig. 2a indicates that there is clear discrimination between the CC probability density functions (PDFs) of catalytic and noncatalytic residues. The data plotted within in Fig. 2 is taken from 283 structurally unique protein chains; meaning no two proteins from a single SCOP superfamily are included. This translates to 96,280 noncatalytic residues and 844 catalytic residues. The PDFs describing datasets parsed by SCOP family (423 proteins) and 80% pairwise sequence identity (568 proteins) are virtually identical to those shown. The average CC values for the catalytic and noncatalytic residues are 0.19 and 0.16, respectively. While Fig. 2a suggests that the most extreme CC scores are not likely to be catalytic, catalytic residues are, on average, more central than noncatalytic residues. A two-sample t-test resolutely confirms that the discrimination between the means is statistically significant (t = 2.0; p = 1.6E-73; sample size = 7,372). Nevertheless, there is appreciable overlap (59.5%) between the two PDFs.

Going further, Fig. 2b compares the PDFs of residues from three accessibility levels to the catalytic residue PDF. The three accessibility levels roughly correspond to the third most buried, middle third and third most exposed residues within the parsed dataset. At each accessibility level, the catalytic residue PDF has a statistically significant increase within its mean value (see Table 1). As discussed above, this result is slightly counterintuitive because the most buried (and thus, most central) residues frequently are not catalytic. Rather, this result demonstrates that catalytic residues are, on average, more central than the top third most buried residues. Again, this result confirms the earlier observations of Amitai. Yet, caution should be exercised when drawing far-reaching conclusions based on this analysis due to the considerable overlap between the distributions. This is especially true in the case of the buried residues, which has 85% overlap with the catalytic PDF.

**Table 1:** Comparison of accessibility to catalytic residue distributions.

**Distribution^1^**	**Average**	**Std. Dev.**	**Number residues**	**t-value^2^**	**p-value**	**Percent overlap^3^**
Catalytic	0.19	0.03	844	---	---	---
Buried	0.18	0.05	6,310	2.0	1.5E-21	84.9
Intermediate	0.16	0.05	5,818	2.0	5.6E-58	70.4
Exposed	0.15	0.05	6,000	2.0	1.2E-101	58.7

^1 ^Accessibility is coarse-grained into three levels, which roughly correspond to the third most buried, middle third and third least buried (exposed) residues. ^2 ^Comparison of each accessibility-filtered PDF to the catalytic PDF; t-value and p-values are from a two-sample T-test comparing each of the accessibility PDFs to the catalytic residue PDF. ^3 ^Percent overlap compares each accessibility level distribution to the catalytic residue distribution, which is calculated over the histogram of sampled data.

### Assessing prediction accuracy of top closeness centrality scores

As stated above, several investigations have examined the prediction accuracy of global centrality metrics; however, none of the previous investigations are on the scale of this report. Nor, have any rigorously controlled for structural redundancy as we do here. Of the previous reports, the largest dataset investigated is 178 proteins [32], which (unlike ours) contained redundant structural folds. Previous investigations use Receiver Operating Characteristic (ROC) plots to examine the balance between true and false positive rates over the entire relationship continuum. A false positive rate greater than 9% is commonly considered; Thibert et al. routinely consider false positive rates ~20%. In this report, we only consider the top N predictions, where N equals 1–5. From a pure prediction point of view, one wants to simultaneously balance sensitivity and specificity. However, when considering experimental realities, we believe our approach has the more relevance because it is less likely to result in huge numbers of false positives that are intractable to test within the lab. The corresponding false positive rate of our predictions is always below 1.6%. Over the entire curve, our ROC plots are virtually superimposable to those within Thibert et al. (results not shown). Unfortunately, a direct comparison to the results within Amitai et al. is impossible since they only provide ROC plots for an integrated sequence conservation/centrality approach. An example ROC plot is provided in Additional file 1.

**Figure 3:**
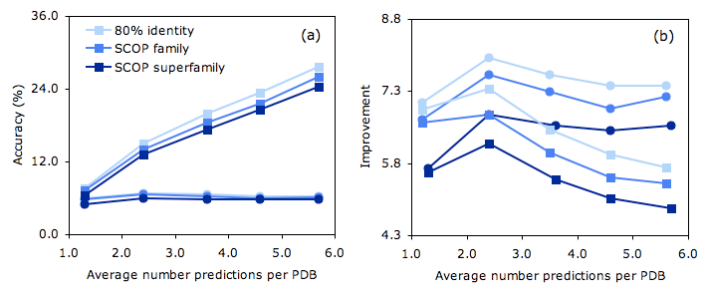
(a) The accuracy for all predictions (circles) and the percentage of PDBs with one correct prediction (squares) is plotted for the three datasets investigated. (b) Improvement normalizes the observed accuracies in (a) by the random expectation.

On average, there are ~5 catalytic residues per protein within the CSA. We use this value as an appropriate upper bound for the number of predictions per protein to consider. The prediction number threshold, T_np_, is defined in order to limit the number of catalytic predictions. At T_np _= 1, only the site with the top CC score is put forth as a prediction; at T_np _= 2, the top two are put forth, and so on. However, the actual number of predictions is routinely greater than T_np _due to ties. Consequently, T_np _is scaled between one and five, and the corresponding average number of predictions per protein is 1.3, 2.4, 3.6, 4.6 and 5.7. The circles in Fig. 3a plot the accuracy (accuracy = number of correct catalytic residue predictions/total number of catalytic residue predictions) against the average number of predictions per protein for each of the three datasets investigated here. The accuracy deviates very little over the full range of T_np_; the average and standard deviation are 5.7% and 0.4%, respectively. When using the common definition of accuracy, (true positives + true negatives)/total number residues, the accuracy corresponds to 98.2%. However, the latter metric provides little useful information as the number of true negatives skews it so heavily. (Note: unless otherwise specified, all reported statistics are from the SCOP superfamily parsed dataset.)

The accuracies described above are from a collapsed dataset of all residues contained within the dataset. Alternately, one may wish to evaluate each protein independently and then average the accuracy values over the total number of proteins contained with the dataset. In each case, only the top T_np _values per protein are considered, the sole difference is how the final average is determined. Below, statistical significance of the methods is demonstrated by calculating p-values from the binomial distribution, which assume independence of trials. However, the assumption of independence is clearly incorrect as residues are connected via the protein chain. Since this assumption is equally wrong in both cases, we chose to primarily focus on the simpler method of averaging across the collapsed dataset. Nevertheless, Table 2 also lists accuracies calculated on the per protein basis. In all cases, the two values are qualitatively similar; however, per protein values are somewhat reduced. Nevertheless, they are still better than the random expectation. Curiously, there is a large variability across the per protein distribution; the standard deviation is ~10%. The two accuracy calculations are statistically equivalent when using the standard deviation of the per protein values as an error estimate; this result is maintained throughout this report. Moreover, the ratio of the collapsed to per protein accuracies is also relatively consistent.

**Table 2:** Evaluation of catalytic site predictions.^1^

**Avg.#/PDB**	**Total accuracy^2^**	**Per PDB accuracy^3^**	**p-value^4^**	**TP & FP rate^5^**	**TP:FP ratio**	**1 correct per PDB^6^**	**1 correct expect^7^**
**(a.) **Raw CC values (no filter)							
1.3	6.0	2.7 (10.8)	2.7E-09	2.1/0.4	6.0	7.6	1.1
2.4	6.8	4.2 (11.6)	2.8E-22	4.9/0.7	7.2	15.0	2.0
3.6	6.5	4.5 (10.6)	2.4E-30	7.0/1.0	6.9	19.9	3.1
4.6	6.3	4.7 (10.0)	2.4E-37	8.8/1.3	6.9	23.4	3.9
5.7	6.3	4.9 (9.6)	9.4E-47	11.0/1.6	6.9	27.6	4.8
**(b.) **Solvent accessibility filter							
1.1	14.2	7.5 (17.4)	2.8E-42	5.3/0.3	18.7	15.9	1.0
2.2	13.0	9.2 (16.8)	7.5E-72	9.7/0.6	16.9	25.4	1.9
3.3	11.1	8.7 (14.7)	6.8E-82	12.2/0.9	14.2	29.3	2.9
4.4	10.8	8.9 (13.2)	4.5E-103	15.8/1.2	13.7	36.7	3.9
5.4	10.4	8.9 (12.4)	2.7E-120 4	18.8/1.4	13.2	41.3	4.8
**(c.) **Residue identify filter							
1.1	22.4	11.3 (21.0)	3.8E-83	8.3/0.3	32.6	23.0	1.0
2.2	19.6	13.5 (19.8)	8.8E-134	14.5/0.5	27.6	35.7	1.9
3.2	17.9	13.8 (18.2)	0.0	19.2/0.8	24.7	42.8	2.8
4.3	17.6	14.3 (17.0)	0.0	25.0/1.0	24.1	50.5	3.7
5.2	16.5	13.9 (156.3)	0.0	29.3/1.3	22.4	56.2	4.7
**(d.) **Combination filter (solvent accessibility + resodue identify)							
1.1	25.2	12.9 (21.8)	0.0	18.6/0.5	39.0	26.1	1.0
2.1	20.7	14.4 (20.7)	0.0	31.0/1.0	30.8	36.7	1.9
3.1	17.9	13.5 (17.1)	0.0	39.9/1.5	26.2	44.2	2.7
4.1	15.9	12.8 (14.6)	0.0	45.4/2.1	21.8	49.8	3.6
5.2	13.9	11.7 (13.1)	0.0	50.0/2.7	18.7	53.0	4.6

^1 ^Statistics describing the accuracy of the accessibility-filtered prediction on the SCOP superfamily dataset. ^2 ^Accuracy is defined as the percentage of correct catalytic residue predictions out of the total number of predictions for the entire collapsed dataset. In all cases, the random expectation is 0.9%. ^3 ^Average value (and standard deviation) of accuracy calculated on a per protein basis. ^4 ^The probability that the null hypothesis is correct calculated from the binomial distribution. ^5 ^The true positive rate is the percent correct of the total number of catalytic residues within the CSA; similarly, the false positive rate is the percent incorrect predictions of the total number of noncatalytic residues. ^6 ^The percent of proteins with at least one correct prediction. ^7 ^The expected percent of proteins with at least one correct assuming a random model.

As stated above, p-values are calculated to test the statistical significance of the hypothesis that CC predictions are better than random (Table 2a). The binomial distribution is used since there are a finite number of trials and the outcome of each is binary. Eq. 2 provides the formula for calculating the p-values.

P(X>k)=∑kn(nk)pk(1−p)n−k, where(nk)=n!k!(n−k)!
 MathType@MTEF@5@5@+=feaafiart1ev1aaatCvAUfKttLearuWrP9MDH5MBPbIqV92AaeXatLxBI9gBaebbnrfifHhDYfgasaacH8akY=wiFfYdH8Gipec8Eeeu0xXdbba9frFj0=OqFfea0dXdd9vqai=hGuQ8kuc9pgc9s8qqaq=dirpe0xb9q8qiLsFr0=vr0=vr0dc8meaabaqaciaacaGaaeqabaqabeGadaaakeaacqWGqbaucqGGOaakcqWGybawcqGH+aGpcqWGRbWAcqGGPaqkcqGH9aqpdaaeWbqaamaabmaabaqbaeqabiqaaaqaaiabd6gaUbqaaiabdUgaRbaaaiaawIcacaGLPaaaaSqaaiabdUgaRbqaaiabd6gaUbqdcqGHris5aOGaemiCaa3aaWbaaSqabeaacqWGRbWAaaGccqGGOaakcqaIXaqmcqGHsislcqWGWbaCcqGGPaqkdaahaaWcbeqaaiabd6gaUjabgkHiTiabdUgaRbaakiabcYcaSiabbccaGiabbEha3jabbIgaOjabbwgaLjabbkhaYjabbwgaLnaabmaabaqbaeqabiqaaaqaaiabd6gaUbqaaiabdUgaRbaaaiaawIcacaGLPaaacqGH9aqpdaWcaaqaaiabd6gaUjabcgcaHaqaaiabdUgaRjabcgcaHiabcIcaOiabd6gaUjabgkHiTiabdUgaRjabcMcaPiabcgcaHaaaaaa@61EB@

In Eq. 2, *n *is equal to the number of predictions put forth, *k *(in the first iteration of the sum) is equal to the number of correct predictions, and *p *represents the random (null) probability. Each step of the sum is calculated from the binomial distribution (*binomdist*) function within Microsoft Excel. Notice that the p-values decrease monotonically (see Additional file 2), despite that the fact that the relative accuracies are not monotonic. In fact, as it is demonstrated below, relative accuracies generally decrease as a function of T_np_. Nevertheless, p-values indicate that the results become more statistically significant at larger T_np _values. This apparent contradiction highlights the true meaning of a p-value. A p-value is the *statistical likelihood *of the null hypothesis being true. It is not an *accuracy *of the method. The smaller the p-value is, the more significant the observed results are. However, statistical significance is intimately related to the number of observations. The more observations of a given difference between an observed and null probability, the more significant it is. The number of predictions put forth put forth at each level increases substantially, whereas the accuracy is only slightly diminished, which is why p-values monotonically decrease as a function T_np_.

The improvement is plotted in Fig. 3b in order to normalize the observed percentages by the random expectation. Improvement is defined as the ratio of observed accuracy to random expectation. The random expectation is simply calculated as the percentage of catalytic to all sites within the dataset, meaning each site has an equal chance of being catalytic. While not overwhelming, the observed accuracies (~6%) are substantially greater than the null model (0.9%). The average improvement over T_np _= 1–5 is 6.4% (standard deviation = 0.4%). The false positive range in Fig. 3a–b is 0.4–1.6%. The false positive rate is calculated as the number of incorrect predictions divided by the total number of noncatalytic residues. True positive rates (number correct divided by the total number of catalytic residues) range from 2.1–11.0%.

While the circles in Fig 3a correspond to overall accuracies, the squares describe the number of proteins with at least one correct prediction per protein. The near linear increase is trivially expected since the number of proteins with at least one correct should increase with the total number of predictions. However, after normalizing for the random expectation in Fig. 3b, the improvement indicates that the rate of new proteins with at least one correct prediction generally decreases as a function of T_np_. Here, the random expectation describes the percentage of proteins with at least one correct again assuming that all sites are equally probable to be catalytic.

**Figure 4:**
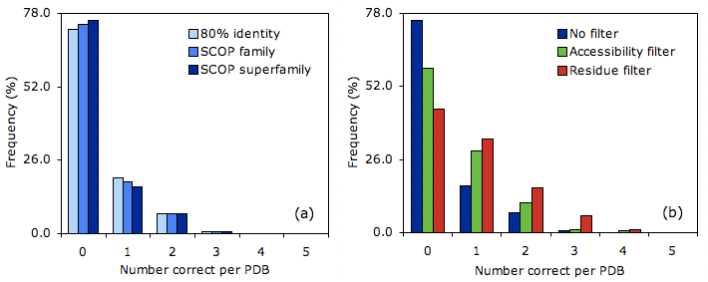
(a) For T_np _= 5, the percentage of PDBs with 0–5 correct predictions are tabulated for each of the three datasets investigated. (b) The same data is plotted for each of the three filtering schemes investigated for the SCOP superfamily parsed dataset. Note: the dark blue series in (a) is exactly the same as in (b).

Fig. 4a tabulates the number of proteins observed with a specific number of correct catalytic residue predictions at T_np _= 5. By far, the most common incidence is zero correct (76%), which corresponds to every prediction within a given protein being incorrect. Approximately 17% of the predictions from the SCOP superfamily parsed dataset have one correct and 7% have two correct. Less than 1% has three correct, and none have four or five correct. Again, there is good agreement between the three datasets, yet (as before) there is a slight accuracy reduction within the smaller datasets.

Throughout this report, we use citrate synthase as an example to discuss the context of the CC results. Citrate synthase is chosen because it nicely demonstrates how the two filters discussed below improve prediction accuracy. Moreover, citrate synthase is an important enzyme in aerobic metabolism; it regulates the pace of the Krebs cycle. The enzyme catalyzes the condensation between the two acetyl carbons from acetyl-CoA and oxaloacetate to form citrate [38]. The reaction is energetically driven by hydrolysis of the thioester bond, which is strongly exothermic, within acetyl-CoA. None of the predictions at T_np _= 5 correspond to catalytic residues. While we are only narrowly using catalytic residues to benchmark the approach, this lack of sensitivity should not be interpreted as a complete failure to provide useful information. Similar to the examples shown in Fig. 1, the five most central residues (Tyr185, Ala186, Phe333, Met335 and Gly336, using 1AJ8 numbering) are all buried deep with the core of the protein; in fact, four are completely inaccessible to solvent. Despite their location within the core, Tyr185 and Phe333 are both clearly important as they structurally contact the catalytic Asp312. Moreover, Phe333 is also contacting the citrate substrate. While all non-protein (HETERO) groups have been stripped from our inputs to make this large-scale analysis tractable, it is evocative that the model is picking residues directly interacting with the substrate, even if they are not catalytic per se. Below it is demonstrated that filtering CC predictions by residue accessibility and/or residue identity substantially improves citrate synthase catalytic residue prediction accuracy.

### Improving prediction accuracy by excluding the most buried residues

Straightforward physical intuition suggests that the most buried residues within the protein are likely to have the highest CC values. Fig. 1 clearly demonstrates this expectation to be correct. However, conventional wisdom also states that most catalytic residues are (at least partially) exposed to solvent [20]. For example, it is very common to find catalytic residues at the bottom of an active residue cleft where they are *partially *obscured from solvent. This is because some exposure to solvent is important for recognition by the incoming substrate. Moreover, water molecules are frequently utilized along the reaction coordinate. As such, it makes sense to filter residue completely inaccessible from solvent from further consideration.

As a first step toward improving CC catalytic residue predictions using solvent accessibility, we begin by asking the question, "*Are the solvent accessibility distributions of catalytic and noncatalytic residues significantly different?*" Additional file 3 clearly shows that the two distributions are very similar. This result justifies the approach because it demonstrates that CC does not simply recapitulate solvent accessibility. Put in other words, CC provides information orthogonal to accessibility. This point is further demonstrated in Additional file 4 that plots accessibility vs. CC for catalytic and noncatalytic residues. Similar to the value reported within Amitai et al. [32], the overall correlation between solvent accessibility and CC is low (R = -0.28). Finally, we use mutual information (MI) to quantify the amount of (in)dependence between the two metrics. The MI between solvent accessibility and CC is 0.011; a value of zero indicates complete independence. Consequently, it makes physical sense to combine the two metrics. This would not be the case if closeness centrality simply reflected solvent accessibility.

We introduce the solvent accessibility threshold, T_sa_, to filter out residues with low solvent accessibilities. All residues with residue solvent accessibility < T_sa _are *a priori *excluded as catalytic residue predictions. Additional file 5 shows two example plots of how accessibility filtering improves prediction accuracy. In all cases, any amount of accessibility filtering significantly increases the prediction accuracies. In the T_np _= 2 example, the maximal relative accuracy occurs at T_sa _= 8 Å^2^, which corresponds to a prediction accuracy of 13.1%. The associated false and true positive rates are 0.6% and 9.8%, respectively. When T_np _= 5, the maximal accuracy (10.4%) occurs at T_sa _= 9 Å^2^. The corresponding false positive rate is 1.4%, and the true positive rate is 18.8%. One might argue that the performance improvement shown here is simply a matter of opening a free parameter with no transferability. In order to test parameter transferability, the parsed dataset was randomly divided into two halves, and the same analysis was performed on each. The resulting ideal thresholds are very close (± 1.0 Å^2^) to each other and to the values for whole dataset. This result confirms the transferability of the identified T_sa _values. Using a fixed T_sa _= 9.0 Å^2^, which is the most common best value observed, Table 2b tabulates the accuracy of the approach at each T_np_. In all cases, the values are greater than the corresponding unfiltered results. Once more, the values from the collapsed and per protein datasets are similar, especially when considering the standard deviation within the per protein values as an error estimate.

Fig. 4b plots the percentage of PDBs with 0–5 correct predictions (T_sa _= 8 Å^2^), which further demonstrates that the solvent accessibility filter improves accuracy. Compared to the unfiltered predictions, there are fewer proteins with zero correct predictions, and more with one or two correct. In the parsed dataset, the improvement for one, two, three and four correct is 12.4%, 3.5%, 0.4% and 0.7%, respectively. In all cases, the p-value for the accessibility-filtered predictions is lower than the corresponding unfiltered results (Additional file 2). In fact, in spite of a global reduction in the number of predictions, the p-value of the accessibility-filtered results is lowered by 33 to 73 orders of magnitude.

As before, we briefly discuss the context of the predictions within the citrate synthase example. Here, the improvement within the catalytic residue predictions is stark. Citrate synthase has three catalytic residues annotated within the CSA. These residues (His223, His262 and Asp312) are structurally proximal to each other and reside within the active residue cleft. Each directly interacts with a carboxyl group of the enzyme's citrate substrate. Recall that the predictions based solely on CC are inaccessible to solvent. On the other hand, all three of the enzyme's catalytic residues are partially exposed to solvent in both the functional dimer and the constituent monomers that are our predictions are based. The monomer exposure of His223, His262 and Asp312 is 34, 52 and 10 Å^2^, respectively. The accessibilities of His223 and His262 within the dimer are slightly reduced, whereas the Asp312 value is unaffected. Based solely on CC (i.e. no filtering), the network model fails to predict either of the catalytic residues; in fact, they only rank order 27^th^, 43^rd ^and 172^nd ^(of 371 residues). Nevertheless, after filtering all residues solvent accessibilities less than 9 Å^2^, His223 and Asp312 are correctly predicted to be catalytic.

As suggested above, sites other than the catalytic residues can also be critical to function [38-40]. Additionally, it is possible that sites not annotated within the CSA might also be catalytic, or at the very least, directly related to functional efficiency. In fact, Russell et al. define ten additional active site residues as being critical to function [37]. In spite of this more liberal definition, none of the remaining three accessibility-filtered predictions (Glu189, Lys219 and Glu228) correspond to sites within the expanded benchmark. Nevertheless, these residues are clearly important, as they are structurally proximal to both catalytic sites. This result is trivially expected due to their sequence proximity to His218; however, the fact that CC, which treats considers each vertex without regard to primary structure, is promising.

### Filtering based on residue identity

While we explicitly avoid alignment and phylogeny data here, it might be possible to improve prediction accuracy by simply filtering out residues that are unlikely to be catalytic based on their innate physiochemical properties. For example, in the neural networkbased prediction approach of Gutteridge et al. [19], it is demonstrated that the single most import element of the input is whether or not the residue being considered is histidine. The second most important element is residue conservation, which is followed closely by whether or not the residue in question is lysine, cysteine, aspartate, glutamate and arginine (in that order). These sequencebased input elements are all more important than a variety of commonsense structural characteristics (i.e. depth, solvent accessibility, cleft information and secondary structure). Consequently, we implement a simple filter based on residue identity here. Any residue that is not histidine, lysine, cysteine, aspartate, glutamate or arginine is excluded from further consideration. We have tried other combinations of residue exclusion, but this provides the best overall results. A comparison of per residue CC values for catalytic and noncatalytic residues is provided in Additional file 6.

The accuracy of the residue identity filtered predictions ranges from 16.5 to 22.4%, which is a substantial improvement over the random expectation of 0.9% (Table 2c). Predicting catalytic residues by residue identity alone provides a second baseline to compare to. In this approach, a prediction is put forth each time one of the six residue types listed above occurs. Using only residue identity results in an accuracy of 2.1%, which is only slightly better than random expectation of 0.9%. Moreover, it is substantially less than the residue identity filtered results, meaning CC substantially improves predictive power over residue identity alone. Like before, the per protein accuracy range is significantly less (11.3 to 14.3%) than the collapsed results. Nevertheless, the main result that the method significantly improves upon the solvent accessibility filtered predictions is clearly conserved.

**Figure 5:**
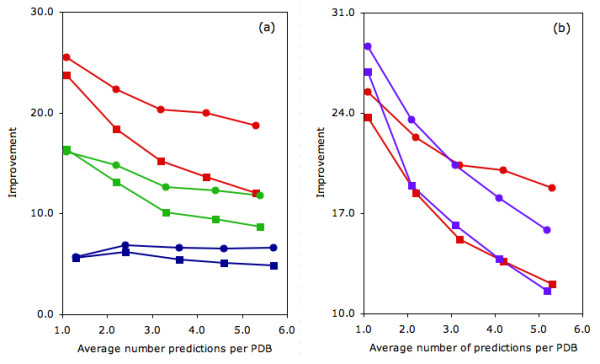
(a) Improvement for all predictions and percent of proteins with at least one correct is plotted for: no filtering (blue), solvent accessibility filtering (green) and residue identity filtering (red). The data presented is from the SCOP superfamily parsed dataset. Symbols are the same as in Fig. 3. In (b), the combination (accessibility + residue identity) filter (magenta) is shown to perform very similar to the residue identity filter alone.

Fig. 5a plots the improvement of the three prediction schemes against T_np_. In all cases, the improvement of the residue identity filtered predictions perform substantially better than the other two schemes. Moreover, an improvement is observed both when considering total prediction accuracy (circles) and the percentage of proteins with at least one correct prediction (squares). As expected, the residue identity filtered p-values are also smaller than the accessibility-filtered ones, despite the fact that there are fewer overall residue filtered predictions. In fact, over the first two values of T_np_, the improvement is 41 and 62 orders of magnitude, respectively. Due to a lack of floating point precision, p-values for the last three are calculated to be exactly zero. Fig. 4b also tabulates the number of PDBs with 0–5 correct residue identity filtered predictions. Again, there is a greater increase in the nonzero bin compared to the accessibility-filtered results. The increase in the number of correctly identified proteins (compared to the unfiltered results) with one, two, three and four correct is 16.6%, 8.8%, 5.3% and 1.1%, respectively. These numbers correspond to an improvement over the accessibility-filtered predictions of 4.2%, 5.3%, 4.9% and 0.4%, respectively.

The residue identity filter decides whether to consider or not consider a particular residue type based on an *a priori *scheme. This is equivalent as saying that the six residues that "pass through" the residue identity filter are equally probable. However, Additional file 6 clearly indicates that this is not reality. As such, it is natural to assume that some sort of fuzzy logic scheme that allows residues to be in the considered or excluded set based on the observed catalytic residue propensities should improve model accuracy. An exhaustive number of schemes were tried using various weighting schemes. For example, three possibilities (from several different considerations) include: (i.) weighting all twenty residues exactly proportional to their catalytic propensity; (ii.) weighting the six from above as equally probable, but scaling of the others; and (iii.) weighting the six from above with exclusion of the remainder. However, no statistically significant improvement over what is reported in Table 2c is found. In the first two examples, the fuzzy model actually does worse since catalytic residues make up such a tiny fraction of the total number of residues. Meaning, any relaxed filtering criteria allows many more noncatalytic (vs. catalytic) residues to be considered; consequently, specificity is lost. Conversely, the best of the trials within the third scheme is statistically indistinguishable from Table 2c.

**Figure 6:**
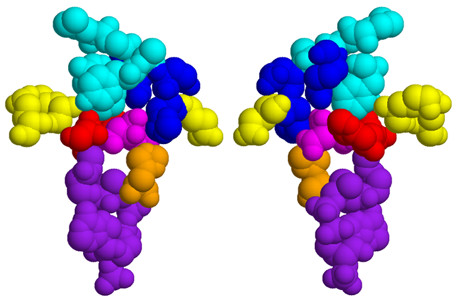
All predictions within citrate synthase, as discussed in the text, are shown in spacefill. Note that they all cluster within the active site region. Coenzyme-A and the citrate substrate are shown in purple and magenta, respectively. The two correctly predicted catalytic residues are shown in red; the unpredicted catalytic residue (His262) is shown in orange. The image on the left, which is rotated 180 degrees from the one on the right, is centered on Asp312 whereas the image on the right is centered on His223. Unfiltered predictions are colored cyan. Except for Lys219, the remaining accessibility and residue identity filtered predictions are colored yellow and blue, respectively. Lys219, which is predicted by both filtering schemes, is colored green (yellow + blue = green).

Filtering based on residue identity results in the following set of predictions (Glu187, His188, Lys219, Asp312 and Arg337) within citrate synthase at T_np _= 5. Asp312 and Lys219 are discussed above, whereas Glu187, His188 and Arg337 are noncatalytic residues within the active site region. As with Phe333, both His188 and Arg337 are directly interacting with the citrate substrate (see Fig. 6). These residues are also interacting with several of the residues identified without filtering. This result, along with those from above, highlights the fact that the three different prediction schemes are identifying citrate synthase residues structurally proximal to the catalytic residues. A similar overall trend is observed when investigating other proteins.

### Combining solvent accessibility and residue identity

Combining both filters together results in slight improvement over the residue identity filter at T_np _= 1–2 (Fig. 5b). At T_np _= 4 and 5, the combination slightly underperforms the residue identity filter, yet the values are still significantly better than the accessibility-filtered results. (At T_np _= 3 the results are virtually identical to the residue identity filtered predictions.) The likely explanation for this result is due to the fact that the filters eliminate similar information. For example, it is trivially expected, due to their propensity to be within the core, that residues eliminated by the accessibility filter will be nonpolar amino acids. Likewise, the residue identity filter always eliminates nonpolar residues from further consideration. As done above with the residue identity filtered results, a baseline without CC is considered. In this instance, any time one of the six considered residue types occurs with a solvent accessibility below 9 Å^2^, a prediction is put forth. This scheme results in an accuracy of 2.5%, slightly better than the 2.1% of residue identity alone, yet nowhere near the accuracies of the combination-filtered CC scores.

### Sensitivity to structural input

It is also important to assess the sensitivity of the CC method to structural input variations. Specifically, ligation state could have a pronounced affect on the observed results. To explore the effects of bound substrate, thirteen randomly chosen structure pairs (with and without ligand) are compared (Table 3). The chosen structures represent a diverse spectrum of protein sizes and SCOP classes. Encouragingly, the average correlation coefficient between CC values for each pair is very high (<R> = 0.970; standard deviation = 0.049), meaning that CC is rather robust to the structural differences. Surprisingly, there is no correlation between CC correlation and pairwise structural RMSD calculated via combinatorial extension [40] (see Table 3). It is also important to consider what is happening between specific catalytic pairs. Comparing the relative rank ordering within each catalytic residue pair reveals that the rank within ligated structures increases 46.2% of the time. Conversely, the rank increases 41.0% of the time within unligated structures; there is no change in the rank 12.8% of the time. Accordingly, there is no systematic performance increase when choosing structures based on the presence or absence of bound substrate.

**Table 3:** Dataset used in comparison of ligated and unligated pairs

**Enzyme^1^**	**Ligated vs. unligated^2^**	**Protein length**	**SCOP class**	**Correl^3^**	**RMSD (Å)^4^**
4-oxalocrotonate tautomerase (1BJP, 4OTB)	1, 2, 0	59	α + β	0.985	0.4
Ribonuclease A (1RBN, 1RSM)	0, 2, 2	124	α + β	0.984	0.6
Xylanase II (1BVV, 1XNB)	1, 1, 0	185	β	0.997	0.2
Trpysin (1A0J, 1UTK)	1, 2, 0	245	β	0.983	1.1
Aminopeptidase (1IGB, 1AMP)	1, 0, 0	291	α/β	0.992	0.6
Phospholipase C (1AOD, 2PLC)	3, 0, 2	294	α/β	0.992	0.2
Deacetoxycephalosporin C synthase (1W2N, 1W28)	0, 1, 0	298	β	0.980	0.5
Chorismate mutase (3CSM, 2CSM)	2, 2, 0	300	α	0.955	1.9
Alginate lyase A1-III (1HV6, 1QAZ)	2, 0, 1	354	α	0.995	0.1
tRNA-guanine transglycosylase (1R5Y, 1PUD)	1, 0, 0	382	α/β	0.814	0.5
Nitric oxide synthase oxygenase (1M9R, 3NOS)	2, 2, 0	480	α + β	0.992	0.2
Luciferase (1BA3, 1LCI)	3, 1, 0	544	Multi.	0.948	0.5
Class I alpha-1;2-mannosidase (1G6I, 1DL2)	1, 3, 0	549	α	0.996	0.1
*Average^3^*				0.970	0.5
*Standard deviation*				0.049	0.5
*Correlation (Correl vs. RMSD)*					-0.17

^1 ^SCOP protein name for each pair examined. The ligated and unligated PDB id's, respectively, are provided in parentheses. ^2 ^The three values (ligated, unligated, tie) tabulate the number of catalytic residues with higher rank ordering between the structural pair. ^3 ^Linear correlation coefficient comparing the CC scores between each structural pair. ^4 ^Pairwise RMSD comparing structure similarity within each pair. Surprisingly, there is no significant correlation between the last two columns.

**Figure 7:**
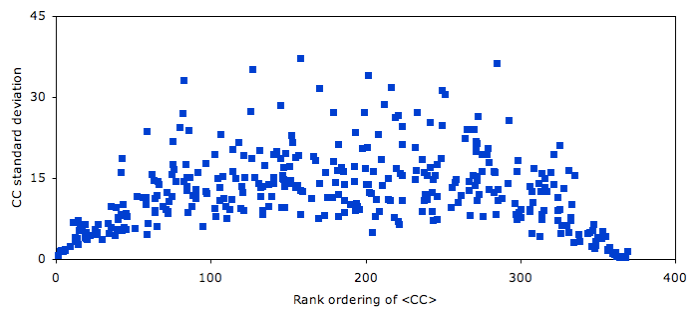
The standard deviation within the CC scores across the structural ensemble is plotted against the average rank ordering of each position for citrate synthase. Residues at the extreme ends of the rank ordering are rather insensitive to the structural variations; however, residues nearer the norm vary significantly. Similar plots are observed in simulations of acetate kinase, triosephosphate isomerase and malate dehydrogenase.

Similarly, we also examined the sensitivity of the CC method to slight structural perturbations. Here, we use molecular dynamics simulations (MD) to "shake up" the structure a small amount (we specifically focus on slight structural perturbations) and compare the resultant CC values. Additional file 7 plots the average CC value + standard deviation for the citrate synthase conformers. The plot clearly demonstrates that CC is fairly robust to these slight structural rearrangements. More importantly, the results concerning catalytic sites predicted after filtering results are overall unchanged. Fig. 7 plots the CC standard deviation vs. the CC rank for each residue within citrate synthase. Residues at the extremes of the CC distribution vary little across the simulation; whereas, residues with CC values near the norm fluctuate much more. Similar results are observed in three other MD simulations. Since catalytic residue CC values are not exclusively at the top end of the distribution, the potential for slight fluctuations to affect prediction accuracy is present (even though it is not observed here). Future work will explore this result more thoroughly.

## Conclusion

This report investigates the ability of CC to predict enzyme catalytic residues from topological descriptions of protein structure. While the most central residues generally correspond to positions within the core, the predictions are substantially better than the random expectation. This result is maintained whether one averages over the collapsed or per protein datasets. Filtering the predictions by solvent accessibility and/or residue identity improves the results considerably. Overall, these results are comparable to those from previous reports [32,33], but have better statistics due to database size and composition. Additionally, we carefully examine the effect of input structure on our predictions. Pairwise comparisons between ligated and unligated structures reveals no clear trend regarding which input is a better choice. Similarly, slight structural perturbations of four protein examples via MD simulation have no observed effect on the overall conclusions.

## Methods

### Dataset

Three different datasets extracted from the manually annotated CSA entries are examined here. The first, which contains 568 PDB files, represents a dataset randomly culled such that no two sequences have greater than 80% sequence identity. The second and third datasets use structural information to randomly distil to nonredundant SCOP [36] families (423 proteins) and superfamilies (283 proteins). In each dataset, a single chain per protein structure is included; however, our analysis of all chains demonstrates that the overall accuracies are generally robust to chain differences (results not shown). All figures shown herein are based on the dataset parsed by SCOP superfamily. However, results for the other two datasets are always similar. This point is typified by Fig. 3 and Fig. 4a, which include data for all three.

### Solvent accessibility

We test the ability of solvent accessibility to improve prediction accuracy by filtering out the most buried residues. Solvent accessibility is calculated using DSSP [42], which is an extremely fast approach. DSSP calculated solvent accessibilities range between 0 to >250 Å^2^. No percent or relative accessibility corrections, which are commonly employed to normalize values by sidechain surface area and to remove backbone considerations, are implemented within DSSP. Nevertheless, the lack of these corrections is not critical here as we are simply trying to identify the residues most excluded from solvent. Theses corrections are more important when quantifying solvent exposure because the maximal accessibility of a large residue (i.e. lysine) is so much greater than that of a small residue (i.e. alanine). Conversely, in our problem, if both residues are maximally buried, the accessibility (with or without the correction) is simply zero in each case.

### Molecular dynamics

Molecular dynamics simulations are employed to generate an ensemble of slightly perturbed structures. The protocol used here is the same as we reported previously in our analysis of sensitivity within calculated pKa values [43]. Canonical ensemble (fixed NVT) *in vacuo *molecular dynamics simulations, as implemented in the Molecular Operating Environment (Chemical Computing Group, Montreal, Quebec, Canada), are used to generate the ensemble of conformers. In each example, the timescale of the simulations is 1 ns, and the timestep is 0.001 ps. Structure sampling occurs every 500 ps. It is obvious that this *in vacuo *simulation protocol is unacceptable to determine realistic aqueous phase dynamics. However, it is adequate for the aims of this work since the simulation is simply used to generate a conformational distribution.

## Abbreviations

Evolutionary trace (ET); Catalytic site atlas (CSA); Closeness centrality (CC); Probability density function (PDF); Receiver operating characteristic (ROC); Mutual information (MI); Molecular dynamics (MD).

## Authors' contributions

EC and DRL contributed equally to the performance of the work described herein. DRL oversaw the research and wrote the manuscript. Both authors have read and approved the final version of the manuscript.

## Supplementary Material

Additional file 1 Supplementary figure 1. This file contains an example ROC curve. Click here for file

Additional file 2 Supplementary figure 2. The probability of the null hypothesis being correct at each T_np _value. Click here for file

Additional file 3 Supplementary figure 3. Probability density functions of the solvent accessibility scores for all catalytic and all noncatalytic sites. Click here for file

Additional file 4 Supplementary figure 4. Scatter plot of residue solvent accessibility vs. closeness centrality. Click here for file

Additional file 5 Supplementary figure 5. The relative accuracy vs. solvent accessibility thresholds is plotted. Click here for file

Additional file 6 Supplementary figure 6. Histogram comparing the catalytic vs. noncatalytic average closeness centrality values for each residue type. Click here for file

Additional file 7 Supplementary figure 7. Average CC value vs. sequence position for citrate synthase. Click here for file
