# Supplementary material for: How accurate and statistically robust are catalytic site predictions based on closeness centrality?
Source: BMC Bioinformatics. 2007 May 11;8:153. doi: 10.1186/1471-2105-8-153 (PMC1876251; doi:10.1186/1471-2105-8-153)
Supplement: Additional file 1 — Supplementary figure 1. This file contains an example ROC curve. [file 1471-2105-8-153-S1.pdf]

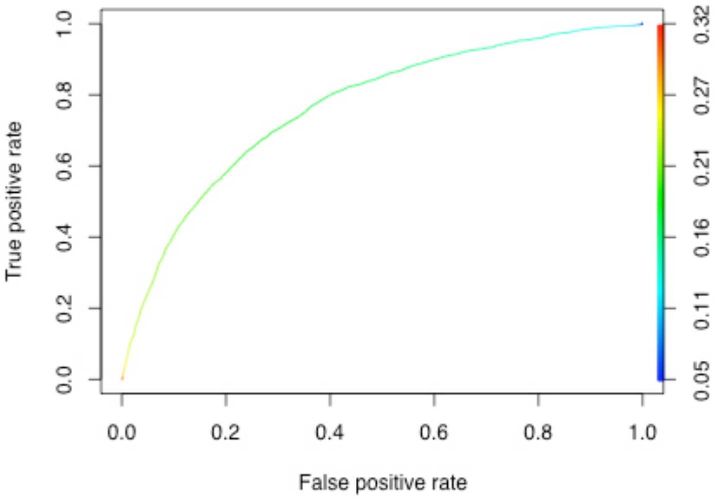

Supplementary figure 1. Receiver Operator Characteristic plot for the unfiltered data. In the ROC plot, sensitivity (TP rate) is plotted against 1-specificity (FP rate). The area under the ROC curve is 0.77; values greater than 0.50 are better than random. This curve is qualitatively identical to the closeness centrality curve in Thibert et al. Unfortunately, a direct comparison to Amitai et al. cannot be made because they only provide plots that include sequence conservation within the predictions. ROC plots are calculated using the Matlab ROC toolkit provided by Dr. Gavin Cawley (see http://theoval.sys.uea.ac.uk/matlab/default.html).
