# Supplementary material for: How accurate and statistically robust are catalytic site predictions based on closeness centrality?
Source: BMC Bioinformatics. 2007 May 11;8:153. doi: 10.1186/1471-2105-8-153 (PMC1876251; doi:10.1186/1471-2105-8-153)
Supplement: Additional file 2 — Supplementary figure 2. The probability of the null hypothesis being correct at each Tnp value. [file 1471-2105-8-153-S2.pdf]

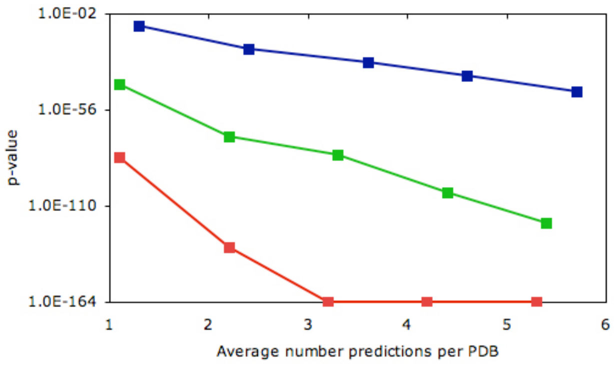

Supplementary figure 2. The probability of the null hypothesis being correct at each T_{np} value; these values correspond to the fourth column in Table 2. Note that due to lack of floating point precision, the third, fourth and fifth p-values of the residue identity filter are calculated to be exactly zero. However, plotting zeros in a logarithmic scale is impossible, so they are arbitrarily plotted as 1.0E-164. The coloring scheme here is the same as in Fig. 4b and Fig. 5a.
