# Supplementary material for: How accurate and statistically robust are catalytic site predictions based on closeness centrality?
Source: BMC Bioinformatics. 2007 May 11;8:153. doi: 10.1186/1471-2105-8-153 (PMC1876251; doi:10.1186/1471-2105-8-153)
Supplement: Additional file 3 — Supplementary figure 3. Probability density functions of the solvent accessibility scores for all catalytic and all noncatalytic sites. [file 1471-2105-8-153-S3.pdf]

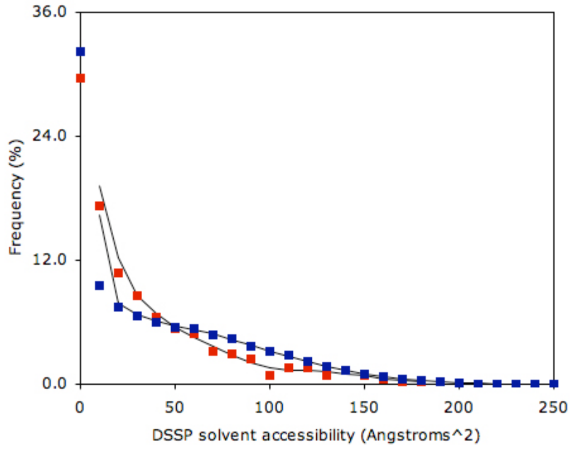

Supplementary figure 3. Probability density functions of the solvent accessibility scores for all catalytic (red) and all noncatalytic (blue) sites. Unlike the closeness centrality distributions shown in Fig. 2, there is significant overlap between the two curves (84%) over the histogram of sampled data. The solid trend line (running average) is provided to guide the eye.
