# Supplementary material for: How accurate and statistically robust are catalytic site predictions based on closeness centrality?
Source: BMC Bioinformatics. 2007 May 11;8:153. doi: 10.1186/1471-2105-8-153 (PMC1876251; doi:10.1186/1471-2105-8-153)
Supplement: Additional file 4 — Supplementary figure 4. Scatter plot of residue solvent accessibility vs. closeness centrality. [file 1471-2105-8-153-S4.pdf]

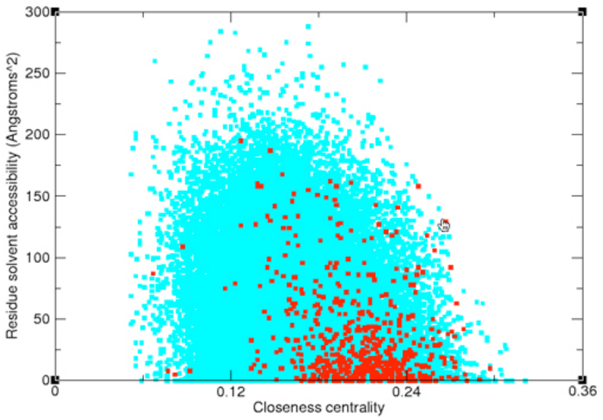

Supplementary figure 4. Scatter plot of residue solvent accessibility ($Å^2$) vs. closeness centrality. This plot clearly demonstrates that the range of the solvent accessibility and closeness centrality values is much greater for the noncatalytic (cyan) residues than it is for the catalytic (red) residues. Note that data point density information is not provided. The overall correlation coefficients between accessibility and CC for all, catalytic and noncatalytic residues are -0.28, -0.27 and -0.29, respectively. Similarly, the mutual information between accessibility and CC is 0.011, which also indicates that the two metrics are virtually independent.
