# Supplementary material for: How accurate and statistically robust are catalytic site predictions based on closeness centrality?
Source: BMC Bioinformatics. 2007 May 11;8:153. doi: 10.1186/1471-2105-8-153 (PMC1876251; doi:10.1186/1471-2105-8-153)
Supplement: Additional file 5 — Supplementary figure 5. The relative accuracy vs. solvent accessibility thresholds is plotted. [file 1471-2105-8-153-S5.pdf]

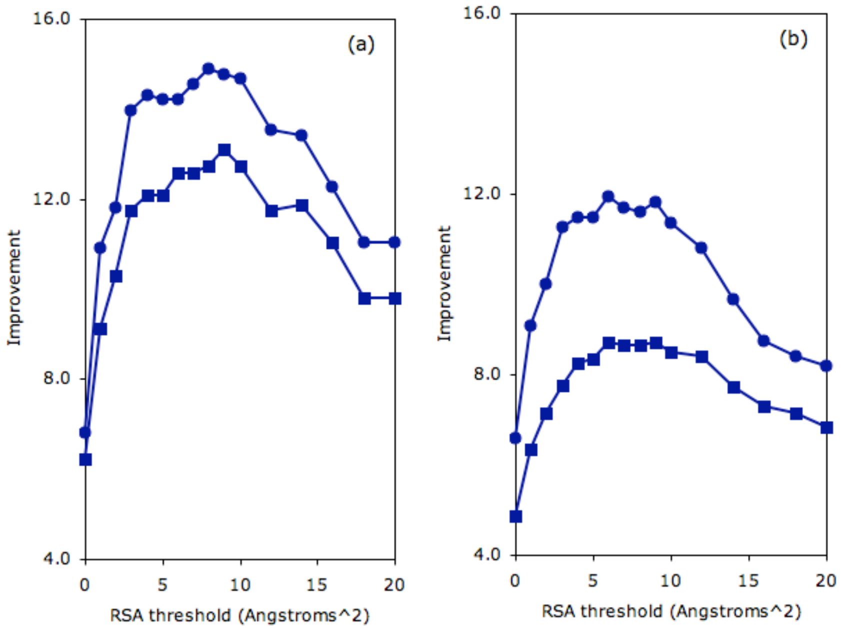

Supplementary figure 5. The relative accuracy vs. solvent accessibility thresholds (T_{sa}) is plotted for (a) $T_{np} = 2$ and (b) $T_{np} = 5$. Circles represent the accuracy for all predictions, whereas squares indicate at least one correct per protein structure. The data presented is from the SCOP superfamily parsed dataset.
