# Supplementary material for: How accurate and statistically robust are catalytic site predictions based on closeness centrality?
Source: BMC Bioinformatics. 2007 May 11;8:153. doi: 10.1186/1471-2105-8-153 (PMC1876251; doi:10.1186/1471-2105-8-153)
Supplement: Additional file 6 — Supplementary figure 6. Histogram comparing the catalytic vs. noncatalytic average closeness centrality values for each residue type. [file 1471-2105-8-153-S6.pdf]

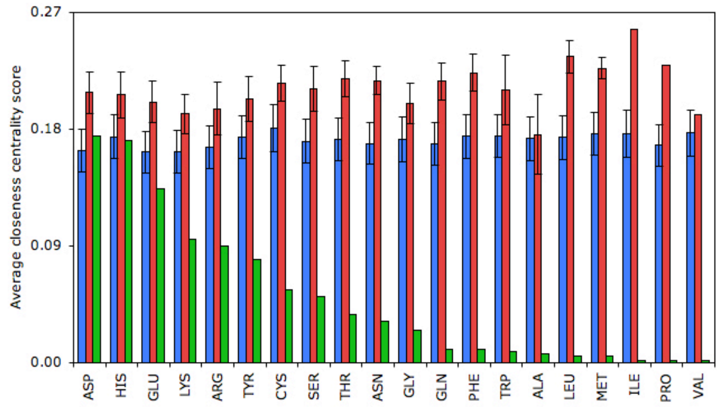

Supplementary figure 6. Histogram comparing the catalytic (red) vs. noncatalytic (blue) average closeness centrality values for each residue type. Error bars represent one standard deviation; no error bars are provided on Ile, Pro and Val because there is only one catalytic example of each. The plot is organized such that the most frequent to least frequent catalytic sites go from left to right. The green series indicates their relative frequency, simply reported on a scale of zero to one.
