# Supplementary material for: How accurate and statistically robust are catalytic site predictions based on closeness centrality?
Source: BMC Bioinformatics. 2007 May 11;8:153. doi: 10.1186/1471-2105-8-153 (PMC1876251; doi:10.1186/1471-2105-8-153)
Supplement: Additional file 7 — Supplementary figure 7. Average CC value vs. sequence position for citrate synthase. [file 1471-2105-8-153-S7.pdf]

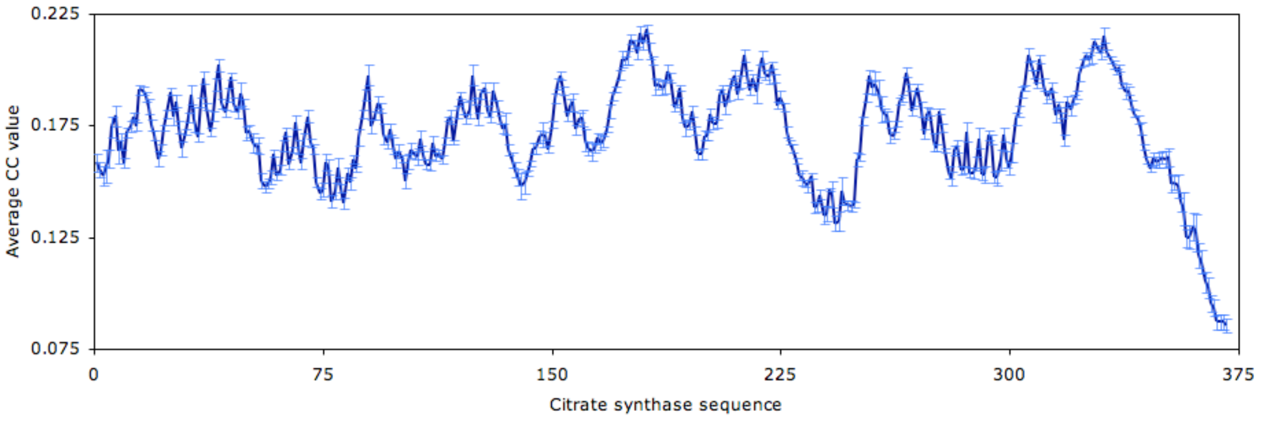

Supplementary figure 7. Average CC value vs. sequence position for citrate synthase. Error bars represent ± one standard deviation.
